# State-of-the-Art Fluorescence Fluctuation-Based Spectroscopic Techniques for the Study of Protein Aggregation

**DOI:** 10.3390/ijms19040964

**Published:** 2018-03-23

**Authors:** Akira Kitamura, Masataka Kinjo

**Affiliations:** Laboratory of Molecular Cell Dynamics, Faculty of Advanced Life Science, Hokkaido University, N21W11, Kita-ku, Sapporo, Hokkaido 001-0021, Japan; kinjo@sci.hokudai.ac.jp

**Keywords:** fluorescence correlation spectroscopy (FCS), image correlation spectroscopy (ICS), number and brightness analysis, super-resolution optical fluctuation imaging (SOFI), transient state (TRAST) monitoring spectroscopy, protein aggregate, neurodegenerative disorder, TDP-43, polyQ, amyloid

## Abstract

Neurodegenerative diseases, including amyotrophic lateral sclerosis (ALS), Alzheimer’s disease, Parkinson’s disease, and Huntington’s disease, are devastating proteinopathies with misfolded protein aggregates accumulating in neuronal cells. Inclusion bodies of protein aggregates are frequently observed in the neuronal cells of patients. Investigation of the underlying causes of neurodegeneration requires the establishment and selection of appropriate methodologies for detailed investigation of the state and conformation of protein aggregates. In the current review, we present an overview of the principles and application of several methodologies used for the elucidation of protein aggregation, specifically ones based on determination of fluctuations of fluorescence. The discussed methods include fluorescence correlation spectroscopy (FCS), imaging FCS, image correlation spectroscopy (ICS), photobleaching ICS (pbICS), number and brightness (N&B) analysis, super-resolution optical fluctuation imaging (SOFI), and transient state (TRAST) monitoring spectroscopy. Some of these methodologies are classical protein aggregation analyses, while others are not yet widely used. Collectively, the methods presented here should help the future development of research not only into protein aggregation but also neurodegenerative diseases.

## 1. Introduction

### 1.1. Protein Folding and Misfolding

Cellular homeostasis is maintained by regulating the quality and quantity of protein molecules [1]. Qualitative and quantitative homeostasis of proteins is referred to as proteostasis or protein homeostasis [2,3,4]. When the cellular proteostasis is disrupted, the cell is affected, resulting in cell death. One possible cause of a proteostasis imbalance is the accumulation of misfolded proteins [4,5]. In a healthy state, molecular chaperones assist protein folding, and misfolded proteins, if produced, are eliminated by a cellular degradation pathway, e.g., proteasome and autophagy systems [6,7]. Dysregulation of the cellular degradation system could result in an increased abundance of misfolded proteins, leading to their accumulation [8,9,10]. Accordingly, protein aggregation is increased and the sequestration of protein aggregates in inclusion bodies (IBs) is often observed [11,12,13]. Therefore, it is important to determine conformational change, early accumulation (oligomerization), and formation of IBs of misfolded proteins in real time. Generally, oligomers and aggregates are difficult to disassemble once they are formed; thus, the aggregation process is essentially a unidirectional reaction. However, the measurement of the dynamics of protein aggregation is complex as various sizes of intermediates are formed during the process; therefore, in order to analyze the specific state of oligomers and aggregates, appropriate methodologies are required. Moreover, protein misfolding is strongly linked to disease-associated amino acid substitutions in proteins [14,15,16]. Therefore, disease-associated protein variants are widely analyzed to determine the aggregation state and cellular toxicity of the aggregates. Fluorescence spectroscopic methodologies play a crucial role in the analysis of the conformational changes of aggregation-prone proteins that are associated with aggregation in solution, as well as in the living cell. Several neurodegenerative disease-associated proteins that are involved in specific issues to be clarified using fluorescence fluctuation-associated methods, as described in the subsequent section.

### 1.2. Transactivation Response Element (TAR) DNA/RNA-Binding Protein 43-kDa (TDP-43)

TDP-43 is an RNA/DNA binding protein that is associated with amyotrophic lateral sclerosis (ALS) and frontotemporal lobar degeneration (FTLD) associated with TDP-43 proteinopathies (FTLD-TDP, previously referred to as FTLD-U) [17,18]. Many ALS-associated missense mutations that cause amino acid substitution in TDP-43, and are involved in the onset and severity of ALS have been identified [19]. TDP-43 contains two RNA/DNA-recognition motifs (RRM1 and RRM2), which mainly recognize single-stranded UG or TG nucleotide repeats, and a C-terminal glycine-rich region (GRR), which includes a prion-like Q/N-rich domain (also called the low complexity sequence domain) [20]. TDP-43 harbors a nuclear localization signal (NLS) between the N-terminal domain and RRM1, as well as a predicted nuclear export signal (NES) in RRM2. Hence, TDP-43 undergoes nuclear-cytoplasmic shuttling; the functions of this protein include mRNA splicing, microRNA processing, and mRNA transport to the cytoplasm [21,22]. A hyper-phosphorylated and ubiquitinated form of TDP-43 accumulates in the IBs in motor neurons of patients with ALS [17,18,23]. Intact TDP-43, as well as its N- and C-terminal fragments (NTFs and CTFs) accumulate in ubiquitin-positive IBs. The primary sequence of TDP-43 contains typical DEVD-like motifs, which are cleaved by caspase 3 [24]. CTFs TDP-43_90–414_ (35 kDa) and TDP-43_220–414_ (25 kDa) are called TDP-35 and TDP-25, respectively [24]. They are prone to aggregation and form cytoplasmic IBs in cultured cells [25,26]. Moreover, TDP-25 tends to be more prone to aggregation than TDP-35, with its aggregation also stimulated by the dissociation of bound RNA [25]. Thus, the relationship between the dysregulation of proteostasis by misfolded TDP-43 and/or TDP-43 CTFs has been implicated in the pathogenesis of ALS. However, the mechanism by which the ALS-associated mutant of TDP-43 is accumulated into the aggregates, as a result of its conformational change, are not known. Thus, real-time observation of the process is important for the elucidation of the underlying processes. Moreover, as TDP-43 often forms IBs and granules in the cell, it is important to distinguish between IBs and granules.

### 1.3. Amyloid Beta/β-Amyloid Peptide (Aβ)

Aβ (or Abeta) is a collective term for peptides consisting of 27–49 amino acids that play a crucial role in the Alzheimer’s disease [27]. The length of the Aβ peptide is associated with the severity of aggregation tendency [28]. These peptides are derived from the amyloid precursor protein (APP), a plasma membrane protein, after cleavage by specific secretases [29]. Aβ is identified as the major components of the amyloid plaques in the brains of Alzheimer’s patients [27]. Aβ forms cytotoxic soluble oligomers and amyloid fibrils in vitro [30]. It is generally believed that soluble Aβ oligomers are the most toxic form, and cause neuronal cell death [31]. The aggregation and oligomeric state of Aβ have been widely analyzed using both fluorescence spectroscopy and other microscopic approaches, including electron microscopy (EM) and atomic force microscopy (AFM) [30,32,33,34]. To detect accumulation of non-fluorescent amyloids in vitro, fluorescent dyes that bind to amyloid structure, such as thioflavin T (ThT), 1-fluoro-2,5-*bis*(3-carboxy-4-hydroxystyryl)benzene (FSB), and 1-bromo-2,5-*bis*(3-carboxy-4-hydroxystyryl)benzene (BSB), are generally used [35,36,37,38]. Moreover, organic fluorescent dyes or green fluorescent protein (GFP) are used for labeling of Aβ in vitro [39,40]. However, methods for the rapid detection of Aβ oligomers in live cells are difficult to establish as the fluorescent dye that fuses to Aβ is often quenched [40]. Therefore, the quenching process of the dye requires further elucidation to enable the development of detection methods with a high sensitivity.

### 1.4. Expanded Polyglutamine (polyQ) Repeat Proteins

PolyQ repeat is present in some proteins, such as huntingtin (Htt), androgen receptor, ataxin, TATA-box binding protein (TBP), cAMP-response element-binding protein (CREB)-binding protein (CBP), etc. [41]. Expanded cytosine-adenine-guanine (CAG) repeats encoding a long (>~40) polyQ tract in the respective proteins have been identified in some neurodegenerative disease patients [42]. The diseases associated with the elongation of the polyQ repeat are hence called polyQ diseases, and include the Huntington’s disease, spinal and bulbar muscular atrophy (SBMA), spinocerebellar ataxia (SCA), and dentatorubral-pallidoluysian atrophy (DRPLA) [42]. Proteins containing expanded polyQ repeat tends to aggregate [41,42,43,44]. Aggregated proteins carrying an expanded polyQ repeat can be sequestrated in insoluble protein deposits (IPOD) in the cytoplasm [11]. The cellular aggregation state of expanded polyQ proteins is maintained by molecular chaperones [45,46,47,48,49,50]. The aggregates contain β-strand structures. Moreover, intermediate-length (27–33) polyQ expansions in ataxin-2 (ATXN2) are associated with an increased risk of ALS, while expansions of >34 repeats cause spinocerebellar ataxia type 2 (SCA-2) [51]. Although fluorescent-labeled polyQ proteins such those are GFP-tagged, emit bright fluorescence in the IBs, it is difficult to detect oligomers in the cytoplasm and nucleus as these oligomers are distributed. Therefore, detection of oligomers, even in a distributed state, is crucial for the development of diagnosis methods.

### 1.5. Importance of Using Fluorescence Fluctuation

The detection of protein aggregation or oligomers in live cell relies on fluorescent dye labeling. Fluorescently labeled molecules are detected by fluorescence microscopy. However, the fluorescence intensity detected by conventional microscopy provides only limited information on the molecular state (e.g., subcellular localization). Hence, methods appropriate for the examination of time scale of changes of the protein state, from conformational change of low-molecular weight species to aggregates, should be employed. AFM or EM are available to detect the shape of protein aggregates; however, the application of such techniques to the real-time observation of accumulation of the misfolded proteins, such as TDP-43, Aβ, and polyQ, in solutions and live cells, is challenging. Therefore, visualization of the protein aggregation state requires the use of advanced spectroscopic techniques.

## 2. Fluorescence Fluctuation-Based Spectroscopic Techniques

### 2.1. Fluorescence Correlation Spectroscopy (FCS)

FCS enables the determination of the diffusion coefficient and the number of molecules by detecting the fluctuations in fluorescence intensity caused by the passage of fluorescent molecules through a detection volume in solution and living cell [52,53]. FCS is widely used to detect the aggregation and oligomers of neurodegenerative disease-associated proteins [25,32,45,47,54]. To generate fluctuation of fluorescence to determine the molecule diffusion state in the detection volume, two important conditions should be met: (i) fluorescent molecules should move in, and pass through, the detection volume; (ii) the number of fluorescent molecules in the detection volume should be low, and their fluorescence counts should be higher than dark counts of detectors. In terms of (i), the passage through the detection volume should be faster than the fluorescence photobleaching rate. In terms of (ii), as the concentration depends on the number of molecules and the detection volume, decreasing the volume allows measurements of relatively highly concentrated molecules. Therefore, immobile or highly concentrated proteins such as those sequestered in the IBs cannot be measured by FCS.

A confocal optical system that includes highly sensitive photo-detectors, e.g., the avalanche photodiode (APD), is widely adopted for the detection of molecular diffusion with a single molecule sensitivity [55,56,57]. When the wavelength of the excitation light is 400–650 nm, and the confocal pinhole size corresponds to the intensity distribution size of the focal center of the airy disk, the detection volume (also called the confocal volume) enters the sub-fL range.

**Figure 1:**
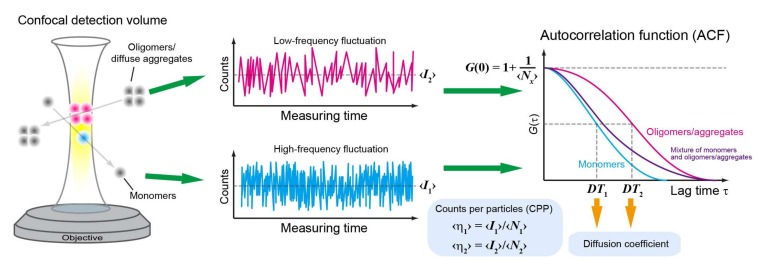
Overview of fluorescence correlation spectroscopy (FCS). Overview of FCS measurement and analysis. ‹*I*_1_› and ‹*I*_2_› represent the average fluorescence intensity of monomers and oligomers/soluble aggregates, respectively. Diffusion times (*DT*_1_ and *DT*_2_) and the number of molecules (‹*N*_x_›) are obtained from the autocorrelation function (ACF) of the fluctuation. Counts per particle (CPP: ‹η_1_› or ‹η_2_›) are calculated using the mean fluorescence intensity during the measurement and the number of molecules. Colors: cyan (monomers), magenta (oligomers/aggregates), and purple (mixture of monomers and oligomers/aggregates; two-components).

For the condition that fluorescence fluctuation to be detected efficiently, the amplitude of the fluctuation gradually increases as the concentration decreases (typically, nM‒µM in the confocal optical system). When the diffusion speed is reduced by increased molecular mass and viscosity, and decreased temperature, the frequency of the fluctuation is reduced (Figure 1). To obtain quantitative information on the fluctuation, the auto-correlation function (ACF) of the fluctuation is calculated, as specified in Equation (1).

(1) $$G\left(\tau \right)=\;\frac{\langle I\left(t\right)\cdot I\left(t+\tau \right)\rangle }{\langle I{(t)\rangle }^{2}}$$

where *G*(*τ*) is ACF; *I*(*t*) is the fluorescence intensities at time *t*; and *τ* is the lag time. Typically, the half-decay time of ACF corresponds to the average residence time of fluorescent molecules in the detection volume. The average number of fluorescent molecules in the detection volume (*N*) can be obtained from the ACF value at time zero, as specified in Equation (2).

(2) $$N=\frac{1}{G\left(0\right)-1}$$

Thus, the concentration of fluorescent molecules (*C*) can be obtained from Equation (3).

(3) $$C=\frac{N}{N_{A}V_{\mathrm{eff}}}$$

where *N*_A_ is the Avogadro constant and *V*_eff_ is the effective detection volume.

In a conventional confocal system, FCS may be used to analyze concentrations of nM‒µM; this upper limitation is due to the detector limit. Various attempts have been made to conduct FCS measurements with higher concentrations than tens of µM (e.g., by decreasing the detection volume using stimulated emission depletion (STED) nanoscopy or by correcting laser fluctuation) [57,58,59]. These improved approaches can be effectively exploited for the determination of weak biomolecular interactions (dissociation constants >~100 µM) [60]. Moreover, FCS that incorporates detectors with high saturation rates would improve the range of the upper limiting concentration. Therefore, improved detectors may be useful for the analysis of fluorescent bright and large protein aggregates.

FCS is highly sensitive to changes in the diffusion state of molecules (Figure 1). The diffusion is reciprocal of the volume/size of the molecule of interest, and hence the size change through aggregation directly influences the diffusion of resulting aggregates. Therefore, it has been used for the determination of the aggregation and oligomeric states of misfolded proteins [25,32,45,47,54]. As mentioned above, since the FCS approach fundamentally cannot be used for the analysis of immobile aggregations, modified FCS is often used for the analysis of the subcellular region, which contains diffused molecules, in living cells or cell lysates [32].

Aβ and polyQ have been classically and traditionally analyzed using FCS [47,61,62,63,64,65]. Although these aggregation-prone proteins were initially regarded as one of the high molecular weight sample for FCS measurement, FCS gradually became used for the analysis of the aggregation process and determining the aggregation-suppressive effect of molecular chaperones and drugs [45,47,66]. Because of the high sensitivity of FCS, oligomers of Htt Q25, which contains a normal-length polyQ repeat and does not form IBs in the cell, have been successfully detected in a cell lysate [67]. Oligomers of expanded polyQ repeat proteins were also detected in live nematode [65]. The polyQ-oligomers can be identified not only by FCS-based diffusion state determinations, but also by measurements of fluorescence intensity distribution of molecules passing through the detection region (photon counting histogram, PCH) [65].

FCS has been used to determine whether the aggregation-prone proteins form oligomers even in a diffused state in a living cell. In fact, the oligomerization properties of diffusing ALS-associated TDP-25 were determined by FCS before they formed IBs in living cells [68]. These studies demonstrate that while, in the cytoplasm and the nucleus, TDP-25 may bind endogenous proteins such as molecular chaperones. Because FCS provides information on the fluorescence counts per second (cps) per particle (counts per molecule/particle; CPM/CPP) (Figure 1), it is possible to directly evaluate the presence of oligomers and soluble aggregates using these parameters. However, a fluorescent protein tagged with misfolded proteins often undergoes quenching. It is thus difficult to directly determine the existence of homo-oligomers based solely on FCS when such quenching occurs. Although quenching of the fluorophore itself can be used to determine the folding state of a protein, even with a single-molecule sensitivity [69], clarification of the cause of fluorophore quenching is somewhat challenging when it is tagged with a protein of interest, because it can be associated with various processes, such as Dexter electron transfer [70,71,72], photo-induced electron transfer (PET) [72,73], the emergence of radicals [72], etc. Moreover, it should be noted that, for intracellular determinations, a slow diffusion state is not necessarily indicative of oligomer formation. During an analysis of tandem oligomers of GFP, an attempt was made to clarify the extinction state by assuming the fluorescence ON state and OFF state of each subunit [74]. Quantification of the quenching efficiency of aggregation-prone proteins should allow direct demonstration of the oligomerization of aggregation-prone proteins without CPM/CPP increase.

On the other hand, a method combining two-photon excitation and FCS to determine protein aggregation without fluorescence-labeling has also been evaluated [75]. In that set-up, changes in the fluorescence lifetime of tryptophan in protein aggregates are monitored in addition to the determination of the increase of diffusion time using FCS.

FCS can be expanded to imaging FCS (ImFCS) [76,77]. In ImFCS, the ACF of each pixel is calculated based on fluctuating fluorescence images captured using a fast frame-rate camera, such as an electron multiplier charge coupled device (EM-CCD) or complementary metal-oxide-semiconductor (CMOS) camera. Thus, unlike FCS, ImFCS is able to reconstitute images of the diffusion kinetics. As the limit of the fast frame rate is conventionally in the ms range, this approach works best for slowly moving molecules such as membrane proteins [78,79]. The development of a camera possessing a faster frame rate with single-photon sensitivity should enable applications requiring fast diffusion state imaging such as the detection of fast diffusing oligomers in live cells.

The upper concentration limit of FCS measurements has been described above. The measurable lower concentration limit of FCS depends on the noise derived from the sample and the detector. Although it is difficult to completely eliminate sample-derived noise, e.g., autofluorescence of a living cell, reduction of the detector noise has indeed been attempted. In FCS, highly sensitive single-photon detectors, such as APD and photomultiplier tube (PMT), have generally been used. The quantum efficiency of APD is higher than that of PMT [80]. Recently, PMT with a photocathode having a high quantum yield (e.g., GaAsP-PMT), and a hybrid photodetector (HPD or HyD) combining a photocathode and an APD have been used as detectors for FCS [81].

**Figure 2:**
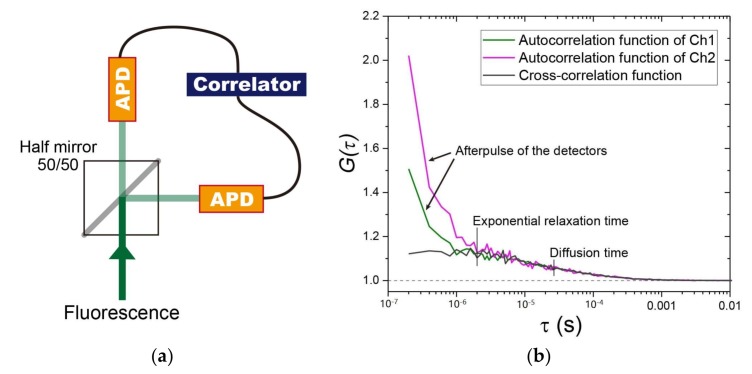
Employing two detectors to reduce autocorrelation curve distortion due to detector afterpulse. (**a**) A fluorescence recoding system using a half-mirror (50/50) and two avalanche photodiodes (APDs). In the system, the denoised autocorrelation function (ACF) is determined based on calculation of the cross-correlation function using the correlator. (**b**) A typical output for an Alexa Fluor 647 dye in Tris-buffered solution. In channels 1 and 2 (green and magenta, respectively), the dominant amplitude of the ACFs within several 10^−7^ s is observed, and is derived from the afterpulse in the detectors. By contrast, the signal derived from the afterpulse can be removed by the cross-correlation function for the two detectors (dark gray). The dashed line indicates the values of 1.0.

Shot noise and afterpulse are the two major types of noise in FCS [82]. These pose a challenge to the detection of the conformational change of protein oligomers and aggregation that cannot be distinguished using translational diffusion measurement; hence, reduction of noise is important for the measurement of rotational diffusion and transient state monitoring spectroscopy of the fluorophores; also see Section 2.5 and Section 2.6. As two types of noises are intrinsic to the detector, they may be removed by separating the fluorescence signal by a half-mirror, measuring the fluorescence using two detectors, and calculating their cross-correlation function [53,83] (Figure 2a,b). However, correlated events shorter than the dead time of the detector (typically < 50 ns) cannot be recorded. As an example, rotational diffusion dependent on the increase of GFP oligomerization was determined using two APDs and calculating their cross-correlation function [56,84]. As the rotational diffusion coefficient is more sensitive to the change of molecular weight than translational diffusion coefficient, the determination of rotational diffusion may be applied for the detection of oligomers of aggregation-prone proteins. Moreover, new single-photon detectors, such as the superconducting nanowire single-photon detectors (SSPDs), which are free from afterpulse, have been developed [84,85]. Such new detectors represent a considerable advance in the analysis of rotational diffusion and other rapid processes, including photochemical reactions.

### 2.2. Image Correlation Spectroscopy (ICS)

ICS was developed as an analog of FCS [86]. By calculating the correlation function based on a fluorescence image generally obtained using confocal laser scanning microscopy (LSM), total internal reflection fluorescence microscopy (TIRFM), or single plane illumination microscopy (SPIM), it is possible to determine the diffusion, transport, and distribution of molecules in a living cell. Although other optical systems may be adopted, LSM, TIRFM, or SPIM enable accurate observation of the fluorescent molecules present on the focal plane with avoidance of noise from scattering light on the glass surface. The spatiotemporal correlation function is defined in Equation (4) [87]:

(4) $$g_{ab}\left(\xi ,\;\eta ,\;\tau \right)=\;\frac{\langle \delta i_{a}\left(x,y,t\right)\delta i_{b}\left(x+\xi ,y+\eta ,t+\tau \right)\rangle }{\langle i_{a}{(x,\;y,\;t)\rangle }_{t}\langle i_{b}{(x,\;y,\;t+\;\tau )\rangle }_{t+\tau }}$$

with the fluctuation in fluorescence, *δi_a_*_or *b*_(*x*, *y*, *t*), given by Equation (5):

(5) $$\delta i_{a\;\text{or}\;b}\left(x,\;y,\;t\right)=i_{a\;\text{or}\;b}\left(x,\;y,\;t\right)-\langle i_{a\;\text{or}\;b}{(x,\;y,\;t)\rangle }_{t}$$

where *i_a_*_or *b*_(*x*, *y*, *t*) is the intensity at pixel (*x*, *y*) in the image recorded at time *t*; and ‹*i*(*x*, *y*, *t*)›*_t_* is the average intensity of the image. The subscripts *a* and *b* refer to the two different colors. In the case of single color detection, *a* is equal to *b*.

Temporal ICS (TICS) is an ICS technique most similar to FCS. It is a method of analyzing the motion state of a molecule from the time correlation function of each pixel when *ξ* and *η* are fixed to zero in Equation (4). When an image sensor, such as EM-CCD or CMOS camera, is used, the rate-determining factor is the time resolution of the detector; thus, the method is not superior to FCS for the determination of the high diffusion coefficient. However, the optical system is not restricted to a confocal optical system; in addition, the fluorescent dye concentration is not limited. Therefore, this method is often used for the analysis of the dynamics of slowly moving membrane proteins using TIRFM, as noise from scattering light on the glass surface may be removed [88,89,90]. Therefore, unlike FCS, TICS may be adopted to determine the speed of slowly moving structures such as granules or IBs including aggregates, in live cells.

Spatial ICS (SICS) can be used for the analysis of the inter-pixel resemblance of fluorescence intensity when τ in Equation (4) is fixed to zero, and it is therefore possible to quantify the average area and distribution for a structure. Although SICS can basically be adapted to the analysis of IBs and granules in a cell, calculated ACF often shows non-Gaussian distribution [91], which poses some difficulties in selecting models determining the structure precisely, and explains why only few studies have been published using SICS. However, the facile quantification of the average size of structures such as IBs or granules represents a great advantage of this method.

### 2.3. Number and Brightness (N&B) Analysis and Photobleaching ICS (pbICS)

**Figure 3:**
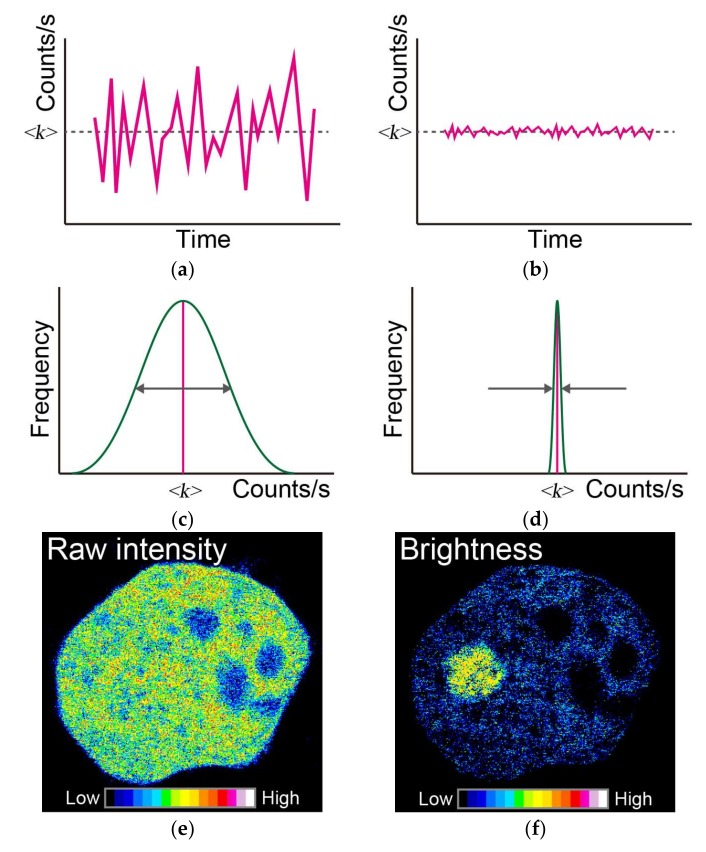
Principles of a typical number and brightness (N&B) analysis. Time-dependent fluctuation of fluorescence intensity in a pixel, such as in (**a**,**b**); in this case, the mean fluorescence intensity is the same as <*k*>, but the variance compared to the mean value is different (comparing between width of double arrow in (**c**) and width between arrows in (**d**)). The distribution of variance is represented in (**c**,**d**). (**e**) Raw image of the recorded fluorescence intensity in a frame. (**f**) Typical image of the brightness of molecules simulated using the N&B approach. Pseudo-color indicates the strength of the raw fluorescence intensities and brightness of molecules (inset color scale in (**e**,**f**)). White arrow in (**f**) indicates the position of aggregates.

To detect the oligomerization of aggregates, other fluctuation methods based on ICS, such as N&B analysis and pbICS, have been adopted [92,93,94,95]. The N&B analysis provides a pixel resolution map of oligomers and aggregates, and the molecular number in a cell, calculated from the average and variance of each pixel in fluorescence images captured on a raster-scanned image obtained with LSM, TIRFM with fast frame-rate cameras, or spinning disk confocal microscope [96,97,98] (Figure 3). Although the optical system is not restricted to a confocal optical system or TIRFM, it is desirable to remove light originating from the non-focal plane. It is basically preferable that the pixel size is not too large with respect to the size of the measuring target. Appropriate concentration range for N&B analysis using confocal microscope has not been strictly proposed and would change depending on the condition of diffusion rate; however, generally, dozens of molar concentrations is used. Although it is a disadvantage that N&B analysis cannot determine diffusion rate, the evaluation of increase in brightness per a particle is important to directly determine oligomerization. N&B analysis can be basically adopted if fluctuation in the pixel is measured, careful verification is required for the pixel dwell time (or frame rate) and total number of frames in which increased brightness per a particle can be actually measured using standard samples such as tandem-oligomers of GFP. The N&B analysis is thus an effective approach for basic studies of the aggregation and oligomers of neurodegeneration-associated misfolded proteins, allowing the analysis of extra- and intra-cellular localization of such aggregation and oligomers, e.g., Htt-polyQ [94,99].

Alternatively, pbICS has been adapted for the detection of Aβ oligomerization [95]; hence, it may be also used for the detection of the oligomeric units of aggregates of various disease-associated proteins. Typically, pbICS would be adapted for the study of immobile aggregates, such as IBs. Although fluorescence recovery after photobleaching (FRAP), as a method based on photobleaching similar to pbICS, can be used for the determination of the proportion of immobile molecules in addition to dynamic properties such as dissociation rate or diffusion coefficient [32,100], quantification of the assembled units using FRAP is challenging. However, it is required that molecules should be larger than submicron scale. Therefore, pbICS may potentially be used more widely in the future.

### 2.4. Fluctuation-Based Super-Resolution Microscopy

Super-resolution microscopy such as stochastic optical reconstruction microscopy (STORM) or photo-activated localization microscopy (PALM) that are based on single-molecule localization measurement, in addition to STED that overcomes the diffraction limited resolution of confocal microscopes as described in Section 2.1, has been widely used in molecular and cellular biology field [101]. The fluctuation of fluorescence may be employed for the reconstruction of super-resolution fluorescence images. A typical application of super-resolution microscopy based on fluorescence fluctuation is super-resolution optical fluctuation imaging (SOFI), which has been evolved for super-resolution imaging of stochastically fluctuating fluorescent dyes [102]. SOFI does not require controlled photo-switching or photo-activation in comparison with other super-resolution microscopy techniques, such as PALM and STORM. The desirable characteristics of SOFI include simplicity, high speed, and low level of light exposure, which provides a probability distribution as an alternative to the moments of the distribution in probability theory and statistics; consequently, several calculation principles have been developed [102,103]. SOFI requires the correlation function-based calculation principle as follows: (i) time-dependent image series with a pixel size smaller than the optical resolution should be acquired; (ii) In the pixel close to the actual center of the emitter, the amplitude of the correlation function becomes higher. On the other hand, in the pixel where the distributions of fluorescence intensity from two independent emitters are overlapped, the amplitude of the correlation function becomes lower; (iii) Thus, integration of the amplitude of autocorrelation function in the pixels can reconstitute super-resolved image. Moreover, calculation of higher order correlation function basically can achieve highly resolved images. To optimize the conditions for achieving super-resolution in SOFI, a software tool has been developed for SOFI under simulated conditions, considering the parameters of the microscope setup and essential properties of the biological sample [104].

### 2.5. Transient-State Monitoring Using FCS

**Figure 4:**
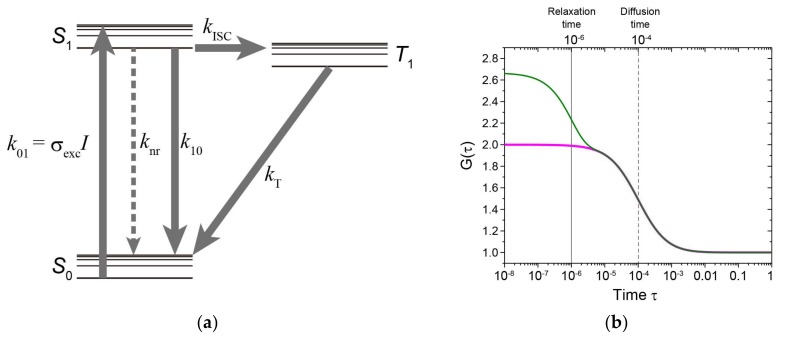

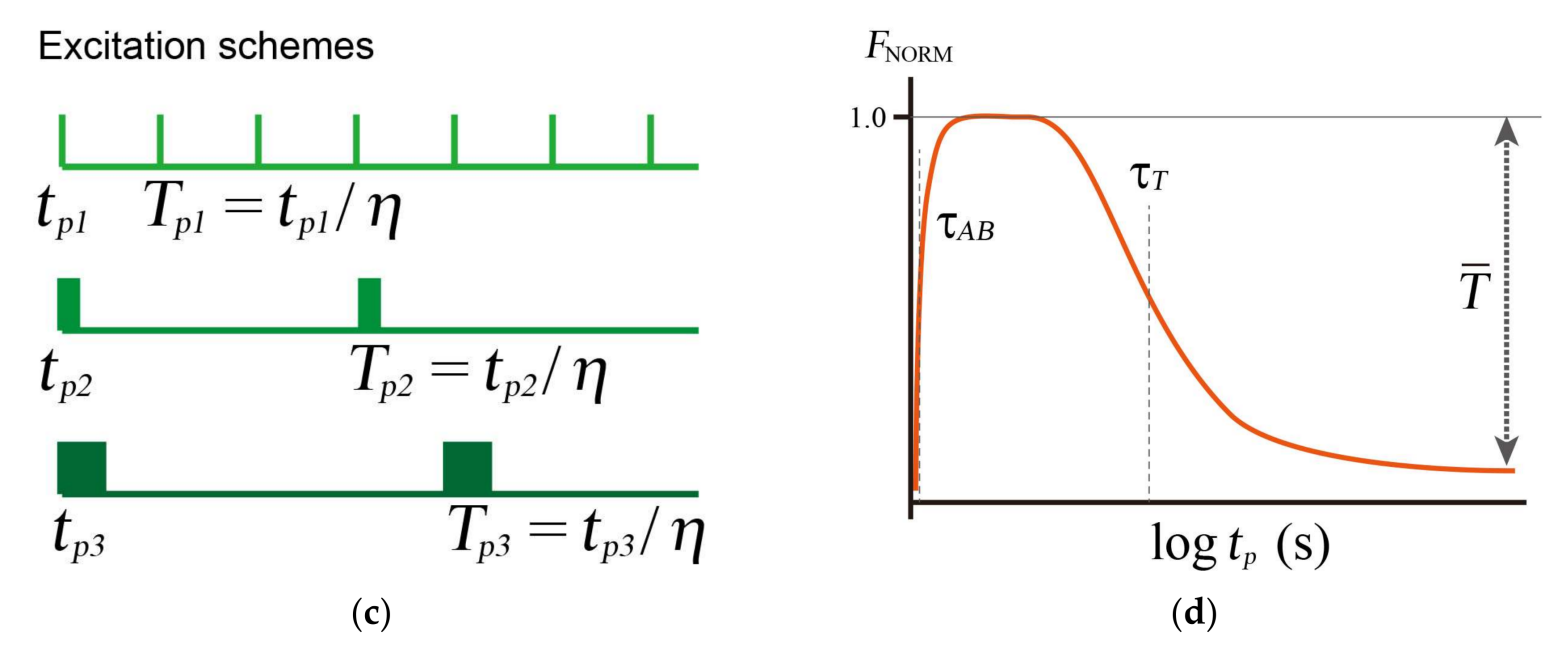
Transient state monitoring with FCS or TRAST. (**a**) Jablonski diagram showing a three-state electronic model, including the ground singlet state (*S*_0_), the first excited singlet state (*S*_1_), and the lowest triplet state (*T*_1_). Rate constants: *k*_01_, for the excitation from *S*_0_ to *S*_1_; *k*_10_, for the relaxation process with irradiation from *S*_1_ to *S*_0_; *k*_nr_, for the relaxation process without irradiation from *S*_1_ to *S*_0_; *k*_ISC_, for the intersystem crossing from *S*_1_ to *T*_1_; and *k*_T_, for the relaxation from *T*_1_ to *S*_0_. *k*_01_ can be written as σ_exc_*I*, where σ_exc_ is the excitation cross-section, and *I* is the excitation intensity. (**b**) Simulated autocorrelation functions *G*(*τ*) as the function of time, *τ*, in a one-component diffusion model (magenta) or one including one-component exponential relaxation (green), where the exponential relaxation time is 10^−6^ s (gray line); the diffusion time is 10^−4^ s (gray dashed line); the component of the exponential relaxation time is 0.4; structure parameter, the ratio between lateral and axial length of the detection volume, is 5; and the number of molecules is 1. (**c**) Excitation schemes of excitation pulses at different pulse times (*t*_p1_, *t*_p2_, and *t*_p3_) and pulse intervals (*T*_p1_, *T*_p2_, and *T*_p3_) during duty cycle, *η*. (**d**) Normalized fluorescence intensity when the transient state cycle is assumed to be the combination of *S*_0_, *S*_1_, and *T*_1_ shown in (**a**) as a change of pulse width. *τ*_AB_, *τ*_T_, and $\bar{T}$ refer to the anti-bunching relaxation time (dashed line), the triplet relaxation time (dashed line), and a steady-state established with a constant population of the lowest triplet state (dark grey two-way arrow), respectively.

Determination of the time dependency of fluorescence intensity enables characterization of fluorophore properties that could not be obtained with steady-state measurements. Fluorophore properties include the lifetime of fluorescence, intersystem crossing, triplet state, radical state, isomerization, etc. (Figure 4a). The relaxation times such as isomerization and fluorescence dynamic quenching via intersystem crossing are usually faster than diffusion time; hence, multi-step decay of the ACF can be observed (Figure 4b). FCS can be adopted to determine these parameters [53,105]. However, to remove the correlated noise in the detector, one should calculate a cross-correlation function between the two detectors or use a detector with a reduced noise [105,106], as described in Section 2.1 (Figure 2). FCS combined with polarization of the excitation light can be adapted to determine molecular orientation, e.g., rotational diffusion [56,84].

The importance of monitoring the transient state of fluorophores basically provides information about the environment of the fluorophores, such as crowding, viscosity, interaction, and conformational change of the molecule of interests. e.g., an increase in viscosity of a solution affects the *cis*-*trans* photo-isomerization rate of iodide carbocyanine fluorophores, such as the cyanine 3 and 5 (Cy3 and Cy5, respectively) dyes [106,107]. Moreover, because such cyanine fluorophores stack onto the end of an RNA molecule [108], the photo-isomerization rate of cyanine dyes would be affected by the properties of the local environment, such as the solvent, or local viscosity and polarity, temperature, and steric hindrance [109,110]. As another example, the fluorophore environment may affect the transition rate of another transient state, including the triplet and radical state, as well as photo-isomerization. Hence, determination of the transient state is an important concept in analyzing the molecule microenvironment.

### 2.6. Transient State (TRAST) Monitoring Spectroscopy Using Time-Resolved Excitation

When the duration of the transient state of the fluorophores is in a range of µs to ms, the decay time of the transient state should overlap with that of the diffusion time in FCS analysis. Therefore, to detect a durable transient state, the principle and approach relying on time-resolved excitation, called TRAST monitoring analysis, was established [53,105,111].

TRAST monitoring spectroscopy measurements employ pulsed excitation, with the mean fluorescence intensity determined for different kinds of excitation pulse widths (Figure 4c). An acousto-optic modulator (AOM) is available and usually used for generation of excitation pulses of different duration. After the onset of excitation, the ground and first excited singlet state equilibrate within ns. When the intersystem crossing probability and oxidation rate is low, the triplet and radical states are slowly generated, and effectively build up after µs to ms, respectively. The population of the first excited singlet state is simultaneously reduced, leading to a decrease of the mean fluorescence. Thus, monitoring the mean fluorescence with different-length excitation pulses can provide information about transient states, such as the triplet and radical state of the fluorophore (Figure 4d). As described in Section 2.5, the transient state of the fluorophores, including *cis*-*trans* photo-isomerization and PET rate, can be altered by the environment. It is therefore convenient to determine this state using TRAST monitoring spectroscopy during aggregate formation because that method may be employed regardless of whether the molecules are mobile or immobile. Analyses of the transient state of tryptophan fluorescence using TRAST monitoring spectroscopy has been reported [112], and therefore the method might be used for determining the conformational changes of proteins. However, it is noticed that various verifications are necessary to link whether the transient state phenomena of the fluorophores are caused by the conformational change of labeling proteins.

Alternatively, the dysregulation of cellular homeostasis, such as redox state and metabolism, is involved in neurodegeneration and aging [113,114,115]. TRAST monitoring spectroscopy can be used to determine the redox and quenched state of the autofluorescent cofactors flavin adenine dinucleotide (FAD) and flavin mononucleotide (FMN) [116]. Thus, it may be adapted for in situ detection of the changes of cellular homeostasis during neuronal cell death in living cells and organs. A conventional confocal LSM may be adopted for TRAST monitoring spectroscopy [117]. Moreover, because the transient state of the fluorophore can be affected by the Förster/fluorescence resonance energy transfer (FRET) [118], TRAST monitoring spectroscopy may be basically used for detecting FRET [119].

## 3. Conclusions

Fluorescence spectroscopy is an important and reliable method for clarifying the mechanism by which protein aggregation dysregulates proteostasis. An appropriate method should be selected based on the object to be analyzed. Although FCS, imaging FCS, and N&B analysis can be used to determine the mobile aggregation and oligomers in solution or in a living cell, immobile components, such as aggregates sequestered in IBs, cannot be analyzed using these approaches. TRAST monitoring spectroscopy can be adopted for the evaluation of conformational changes and the microenvironment of fluorophores tagged with aggregation-prone proteins in such immobile components. This is because TRAST monitoring spectroscopy makes it possible to determine the change in the mean fluorescence intensity according to the excitation time regardless of the mobile or immobile state of the molecules of interests. ICS is useful when determining the shape and distribution of IBs, including protein aggregation, and transporting dynamics of the slowly moving structure in living cells. Fluorescence fluctuation recordings can be employed to improve the current analytical approaches, such as SOFI as super-resolution microscopy. Consequently, methods including FCS, imaging FCS, ICS, N&B analysis, SOFI, and TRAST monitoring spectroscopy have been already used to a certain extent to molecular and cellular biology; however, their importance in facilitating new insights into neurodegenerative diseases is bound to grow in the future.

## Abbreviations

FCS	Fluorescence correlation spectroscopy
ICS	Image correlation spectroscopy
pbICS	Photobleaching ICS
N&B	Number and brightness
SOFI	Super-resolution optical fluctuation imaging
TRAST	Transient state
ACF	Autocorrelation function
APD	Avalanche photodiode
PMT	Photomultiplier tube
LSM	Laser scanning microscopy
IBs	Inclusion bodies
ALS	Amyotrophic lateral sclerosis
polyQ	Polyglutamine
